# Flurbiprofen in patient-controlled intravenous analgesia and the risk of postoperative nausea and vomiting after gynecologic laparoscopy: a retrospective case-control study

**DOI:** 10.3389/fphar.2026.1785675

**Published:** 2026-03-19

**Authors:** Jingru Chen, Qi Liu, Jingyuan Chen, Manting Xie, Yiru Rao, Pingqian Wang, Wenqi Huang, Qiulan He

**Affiliations:** 1 Department of Anesthesiology, First Affiliated Hospital of Sun Yat-sen University, Guangzhou, Guangdong, China; 2 Department of Anesthesiology, The First Affiliated Hospital of Harbin Medical University, Harbin, Heilongjiang, China

**Keywords:** flurbiprofen, laparoscopic gynecologic surgery, pain, patient-controlled intravenous analgesia, postoperative nausea and vomiting, postoperative period

## Abstract

**Aims:**

To measure the possible association between flurbiprofen-containing patient-controlled intravenous analgesia (PCIA) and an increased risk of postoperative nausea and vomiting (PONV) following gynecological laparoscopic surgery.

**Methods:**

The retrospective study was performed on 2,430 patients who underwent a gynecological laparoscopic surgery between April 2021 through May 2022. Perioperative data were extracted from electronic medical records. Multivariable logistic regression was used to identify independent risk factors for PONV, supported by propensity score matching (PSM) and subgroup analyses. A directed acyclic graph (DAG) was constructed to guide confounder selection.

**Results:**

Among 2,430 eligible patients, PONV occurred in 27.7% (554/2000) of patients with flurbiprofen-containing PCIA *versus* 23.0% (99/430) without (absolute risk difference: 4.7%; number needed to harm [NNH] = 21). Multivariable analysis confirmed an independent association between flurbiprofen use and higher PONV risk (adjusted odds ratio [OR] = 1.414, 95% confidence interval [CI] = 1.042–1.918, p = 0.026). Subgroup analysis showed a particularly elevated risk in patients >60 years and those receiving hydromorphone-based PCIA. Notably, despite lower opioid consumption, patients with flurbiprofen had higher PONV incidence.

**Conclusion:**

Flurbiprofen-containing PCIA was associated with a modest yet clinically meaningful increase in PONV, especially when combined with hydromorphone. These findings challenge the assumption that NSAIDs are universally protective against PONV and underscore the importance of tailoring multimodal analgesia strategies according to patient risk profiles.

## 1 Introduction

Among the various complications of anesthesia, postoperative nausea and vomiting (PONV) has become a major problem with reported incidence ranging from 20% to 30% in the general surgical population to over 80% in high-risk patients (Gan et al., 2020). PONV not only causes patient discomfort and dissatisfaction, but also leads to dehydration, wound issues, unanticipated hospital admissions, and increased healthcare costs from prolonged recovery (Fortier et al., 1998; Gold et al., 1989; Gillmann et al., 2019; Colvin et al., 2019; Ao et al., 2025). Therefore, reducing PONV is still a priority for perioperative care and patient safety.

Established patient-related risk factors for PONV include female sex, non-smoking status, a history of motion sickness or prior PONV, and postoperative opioid use (Gan et al., 2020). In enhanced recovery protocols, minimizing perioperative opioids *via* multimodal analgesia is a key strategy to lower baseline PONV risk (Gan et al., 2020; Colvin et al., 2019; Ao et al., 2025). Analgesic strategy has become a focal point in PONV management. Non-opioid analgesics, especially nonsteroidal anti-inflammatory drugs (NSAIDs), are widely incorporated into multimodal pain control to reduce opioid requirements (Doleman et al., 2021; Wick et al., 2017).

Flurbiprofen, an injectable NSAID, is one such adjunct increasingly used in patient-controlled intravenous analgesia (PCIA) protocols. In China and other Asian settings, flurbiprofen is commonly administered as part of opioid-sparing PCIA regimens (Song et al., 2022; Zhang et al., 2025). Its pharmacological actions (COX inhibition and anti-inflammatory analgesia) effectively reduce postoperative pain and opioid requirements, contributing to enhanced recovery (Liu et al., 2024). The direct influence of flurbiprofen on PONV, however, remains unclear. While NSAIDs-based analgesia could theoretically mitigate PONV by minimizing opioids, flurbiprofen may also impact gastrointestinal motility or interact with central pathways in ways that might offset its opioid-sparing benefit.

Despite that ERAS protocols advocate for opioid-sparing anesthesia and multimodal analgesic strategies (Macintyre et al., 2022), these recommendations have not been consistently or effectively reflected in real-world PCA practices. Several studies have reported that patients receiving postoperative PCA may actually consume higher total doses of opioids compared with those managed with non-PCA approaches, such as targeted regional techniques (Black et al., 2023; McNicol et al., 2015). Conventionally, the combination of NSAIDs was assumed to decrease perioperative opioid consumption. However, in the clinical reality of China, where PCA remains the predominant postoperative analgesic technique, overall opioid consumption has often not decreased as expected. This indicates a potential practice pattern where the non-opioid is superimposed rather than utilized for true opioid substitution. Therefore, the influence of combining NSAIDs on analgesia-related adverse effects requires attention.

To date, no large-scale study has specifically examined whether adding flurbiprofen to PCIA opioids alters PONV outcomes. We therefore conducted a retrospective case-control analysis to determine whether flurbiprofen-containing PCIA is independently associated with PONV risk in this high-risk surgical population, and to explore any patient subgroups in which this effect may be pronounced.

## 2 Materials and methods

### 2.1 Study design

The present study was a retrospective case-control study and was approved by Independent Ethics Committee for Clinical Research and Animal Trials of the First Affiliated Hospital of Sun Yat-sen University (protocol#2022-011). Strengthening the Reporting of Observational studies in Epidemiology (STROBE) reporting guideline was followed. As the study was a retrospective analysis, sample size calculation was not performed.

### 2.2 Population

The retrospective review was performed on patients who underwent a gynecological laparoscopic surgery between 1 April 2021 through 11 May 2022 in the First Affiliated Hospital of Sun Yat-sen University. Inclusion criteria comprised: scheduled to undergo gynecological laparoscopic procedures under general anesthesia, American Society of Anesthesiologists (ASA) physical status class I-III, normal hepatic and renal function and absence of major comorbidities, preoperative fasting duration ≥8 h for solids and ≥2 h for liquids, actual PCIA using duration exceeding 24 h. Pregnant women and the patients who underwent emergency surgery were excluded.

### 2.3 Anesthesia and pain management

All enrolled patients received either combined intravenous-inhalational general anesthesia or total intravenous anesthesia. Anesthesia induction was accomplished with propofol 1.5–2 mg/kg, sufentanil citrate 0.3–0.5 μg/kg and rocuronium bromide 0.6 mg/kg or cisatracurium besylate 0.15 mg/kg. Intraoperative analgesia regimen was target-controlled infusion (TCI) of propofol 2–3 g/mL and remifentanil 2–4 ng/mL. Flurbiprofen 50 mg was administered intravenously after the start of the procedure. Intermittent intravenous injection of rocuronium or cisatracurium was conducted to maintain muscle relaxation. If the anesthesia method was combined intravenous-inhalational anesthesia, 1%–1.5% sevoflurane was inhaled.

The PCIA regimens were determined by the chief anesthesiologists based on individual patient needs, resulting in heterogeneity in opioid type, background infusion rates, and use of antiemetics. The most comprehensive formulations included opioids, flurbiprofen, and antiemetics, but not everyone had all these ingredients and opioid types and dosages were not standardized. To address this variability analytically, we converted all opioid dosages to morphine milligram equivalents (MME) for both intraoperative and postoperative periods.

### 2.4 Data collection

The outcome of this study was the incidence of PONV within 48 h after surgery. The degree of nausea and vomiting was evaluated by the simplified nausea verbal descriptive scale (NVDS), which was 0 if there was no nausea or vomiting, one if there was nausea, and two if there was vomiting. Patients with an NVDS score greater than 0 were classified into the PONV group. It should be noted that this scale does not differentiate between the severity of nausea *versus* vomiting, the timing of onset, or the requirement for rescue antiemetics. Postoperative pain was assessed using the numeric pain rating scale (NPRS), from 0 to 10, with 0 indicating no pain, one to three indicating mild pain, four to six indicating moderate pain, and 7–10 indicating severe pain. PONV and pain scores were assessed and recorded by the pain ward rounds specialists on the day of surgery, the first day after surgery and the second day after surgery. In addition, the baseline demographic data and perioperative data of participants including age, body mass index (BMI), smoking status, operation time, PCIA regimen and else were also collected through the electronic medical record. The dosage of opioid use during operation and within PCIA was converted to morphine milligram equivalent (MME) for analysis.

### 2.5 Statistical analyses

All statistical analyses were conducted using the IBM SPSS Statistics (version 26.0, IBM Corp.) and R (version 4.3.1, R Foundation for Statistical Computing). Sample size adequacy was confirmed through *post hoc* power analysis, which demonstrated >80% power to detect the observed effect size (OR = 1.414) at α = 0.05. Missing data were handled using multiple imputation. Normal variables were presented as mean ± standard deviation. Non-normally distributed measurements were expressed as medians (interquartile ranges) or count (%) as required. Student’s t-test (2-sided) and Wilcoxon’s test were used to compare statistical difference between groups. Pearson chi-square test or Fisher’s exact test was used to analyze ordinal or categorical variables. In order to analyze the risk factors of PONV within 48 h after surgery, univariate regression analysis was performed on all relevant variables according to the outcome and a directed acyclic graph was constructed to screen out confounding factors. Then perioperative indicators with p < 0.05 in the results of univariate regression analysis and other confounding factors were selected as covariates included in the multivariate logistic model. With whether PONV occurred within 48 h after surgery as the dependent variable, the odds ratio (OR) and 95% confidence interval (CI) of the study factors was calculated to obtain the corresponding independent risk factors. In addition, PSM and stratified analysis were conducted as sensitivity analyses to ensure the robustness of the study.

### 2.6 AI declaration

We used ChatGPT-4.0 solely for language polishing. All AI-generated text was reviewed and edited by the authors to ensure accuracy and consistency with the study’s data. AI tools were not used for study design, data collection, data analysis, interpretation, or conclusions. The authors retain full responsibility for the content.

## 3 Results

### 3.1 Patient characteristics

**FIGURE 1:**
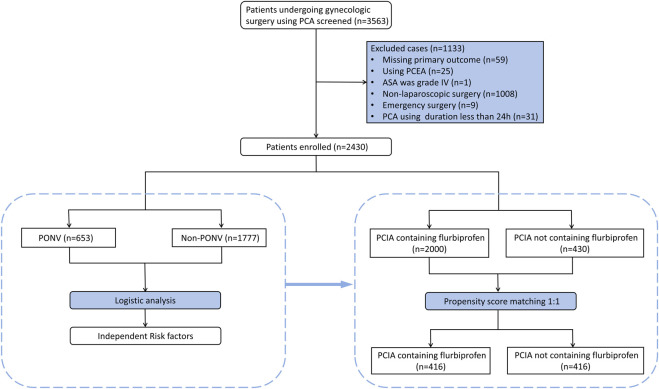
Flow diagram detailing the selection process for patients included in this retrospective analysis. PCA, patient-controlled analgesia; PCEA, patient-controlled epidural analgesia; ASA, American Society of Anesthesiologists; PONV, postoperative nausea and vomiting; PCIA, patient-controlled intravenous analgesia.

The study retrospectively collected all 3,563 patients receiving PCA after gynecological surgeries from 1 April 2021 to 11 May 2022. Of the 3,563 patients recruited into the study, 1,133 patients were excluded owing to missing primary outcome (n = 59), using patient-controlled epidural analgesia (PCEA) (n = 25), ASA class IV physical status (n = 1), non-laparoscopic surgery (n = 1,008), emergency surgery (n = 9) and PCA using duration less than 24 h (n = 31). Ultimately, 2,430 patients were included in the final statistical analysis (Figure 1). Of these, 2000 patients received PCIA containing flurbiprofen, while the remaining 430 patients received flurbiprofen-free PCIA regimens. A 1:1 propensity score matching (PSM) was implemented to balance potential perioperative confounders between the two groups, subsequent comparative analysis and subgroup analysis were performed on the matched cases.

Demographic data are presented in Table 1. The groups were similar in most baseline characteristics including age, ASA, fasting time, operative time, intraoperative MME, use of sevoflurane and so on. The median age of all patients was 45 (IQR, 37–52) years. Compared to patients without PONV, those experiencing PONV demonstrated significantly lower body weight [56.0 (50.0–62.0) kg vs. 57.0 (52.0–63.0) kg; p < 0.001] and lower BMI [22.21 (20.04–24.75) kg/m^2^ vs. 22.72 (20.78–24.98) kg/m^2^; p < 0.001]. Additionally, patients with history of motion sickness or nausea and vomiting exhibited higher PONV incidence (29.71% vs. 22.85%; p < 0.001). Only 17 of the 2,430 patients were smokers in the present study.

**TABLE 1:** Clinical and demographic characteristics and surgery outcomes.

Variables	Total	Postoperative nausea and vomiting		*p* value
​	n = 2,430	Yes (n = 653)	No (n = 1777)	​
Age (yr)	45 (37, 52)	45 (36, 51)	45 (37, 52)	0.189
Weight (kg)	56.9 (51.0, 63.0)	56.0 (50.0, 62.0)	57.0 (52.0, 63.0)	**<0.001**
Height (cm)	158 (155, 162)	158 (155, 161)	158 (155, 162)	0.257
BMI (kg/cm^2^)	22.6 (20.6, 24.9)	22.2 (20.0, 24.8)	22.7 (20.8, 25.0)	**<0.001**
History of motion sickness or nausea and vomiting	600 (24.69%)	194 (29.71%)	406 (22.85%)	**<0.001**
History of smoking	17 (0.70%)	3 (0.46%)	14 (0.79%)	0.434
Hemoglobin (g/L)	121.0 (107.0, 130.0)	122.0 (109.0, 130.5)	121.0 (106.0, 130.0)	0.158
Albumin (g/L)	39.0 (37.0, 42.0)	39.0 (37.0, 42.1)	39.0 (37.0, 42.1)	0.771
Fasting time (h)	14.08 (11.75, 16.30)	14.17 (11.78, 16.58)	14.07 (11.75, 16.19)	0.258
ASA, n (%)	​	​	​	0.681
I	385 (15.90%)	103 (15.80%)	282 (15.93%)	​
II	1824 (75.31%)	497 (76.23%)	1,327 (74.97%)	​
III	213 (8.79%)	52 (7.98%)	161 (9.10%)	​
Intraoperative sevoflurane	2004 (82.47%)	1,459 (82.10%)	545 (83.46%)	0.436
Intraoperative antiemesis	814 (33.50%)	647 (36.41%)	167 (25.57%)	**<0.001**
Involving digestive tract	238 (9.79%)	165 (9.29%)	73 (11.18%)	0.164
Operative time (h)	2.70 (2.00, 3.92)	2.67 (1.95, 3.94)	2.82 (2.08, 3.84)	0.121
Intraoperative hypotension	2019 (83.09%)	1,471 (82.78%)	548 (83.92%)	0.506
Intraoperative MME (mg/kg)	2.75 (1.89, 3.99)	2.87 (1.96, 4.06)	2.71 (1.86, 3.92)	0.190
Intraoperative fluid replacement volume (mL/kg)	24.49 (18.41, 33.33)	26.09 (19.35, 34.74)	24.00 (18.18, 32.69)	**0.007**
Intraoperative blood loss (mL/kg)	0.81 (0.33, 1.73)	0.89 (0.38, 1.85)	0.77 (0.31, 1.67)	**<0.001**
Intraoperative urine output (mL/kg)	1.08 (0.00, 4.73)	1.06 (0.00, 4.74)	1.10 (0.00, 4.72)	0.788
Intraoperative net fluid balance (mL/kg)	20.83 (15.15, 28.00)	22.03 (15.59, 30.00)	20.45 (14.96, 27.58)	**0.005**
P_ET_CO_2_ < 30 mmHg (minutes)	20 (5, 70)	15 (5, 60)	20 (5, 70)	**0.037**
P_ET_CO_2_ > 40 mmHg (minutes)	0 (0, 5)	0 (0, 5)	0 (0, 5)	**0.011**
PCIA containing flurbiprofen	2000 (82.30%)	554 (84.84%)	1,446 (81.37%)	**0.047**
PCIA containing antiemetics	380 (15.64%)	291 (16.38%)	89 (13.63%)	0.099
Background MME of PCIA (μg/kg/h)	6.38 (0.00, 11.50)	6.77 (0, 12.41)	6.32 (0, 11.18)	0.585
Total MME of PCIA (μg/kg)	328.3 (157.9, 651.9)	237.8 (110.1,487.2)	359.8 (181.7,688.7)	**<0.001**
Postoperative LHS (days)	5 (3.6)	5 (4.6)	5 (3.6)	0.195
NRSr, M (Q1, Q3)	0 (0.1)	1 (1.2)	0 (0.0)	**<0.001**
NRSm, M (Q1, Q3)	2 (1.2)	2 (2.3)	1 (0.2)	**<0.001**

Values given as Mdn (IQR) or n (%).

Abbreviations: BMI, body mass index; ASA, american society of anesthesiologists; MME, morphine milligram equivalents; Intraoperative net fluid balance = Intraoperative fluid replacement volume - Intraoperative blood loss - Intraoperative urine output, expressed per kilogram of body weight; P_ET_CO_2_, End-tidal carbon dioxide partial pressure; PCIA, patient-controlled intravenous analgesia; LHS, length of hospital stay; NRSr, numeric rating scale at rest; NRSm, numeric rating scale during movement.

Intraoperatively, the PONV group received more dexamethasone and 5-HT_3_ receptor antagonist intravenously as antiemesis (36.41% vs. 25.57%; p < 0.001) and had higher intraoperative fluid administration [26.09 (19.35–34.74) mL/kg vs. 24.00 (18.18–32.69) mL/kg; p = 0.007], greater blood loss [0.89 (0.38–1.85) mL/kg vs. 0.77 (0.31–1.67) mL/kg; p < 0.001], and higher fluid balance [22.03 (15.59–30.00) mL/kg vs. 20.45 (14.96–27.58) mL/kg; p = 0.005]. They also had shorter periods of end-tidal CO_2_ < 30 mmHg [15 (5–60) min vs. 20 (5–70) min; p = 0.037] and longer periods of end-tidal CO_2_ > 40 mmHg [0 (0–5) min vs. 0 (0–5) min; p = 0.011].

With respect to analgesic regimens, the use of flurbiprofen-containing PCIA was slightly more frequent among patients with PONV compared with those without PONV (84.84% vs. 81.37%; p = 0.047). The cumulative opioid consumption within PCIA was significantly lower in the PONV group [237.8 (110.1–487.2) µg/kg vs. 359.8 (181.7–688.7) µg/kg; p < 0.001]. However, the numeric rating scale were higher in patients with PONV, both at rest [1 (1–2) vs. 0 (0–0); p < 0.001] and during movement [2 (2–3) vs. 1 (0–2); p < 0.001]. In the flurbiprofen group, the dosage of flurbiprofen was 35.11 (16.24–61.64) mg.

### 3.2 Multivariate logistic regression analysis of PONV

**FIGURE 2:**
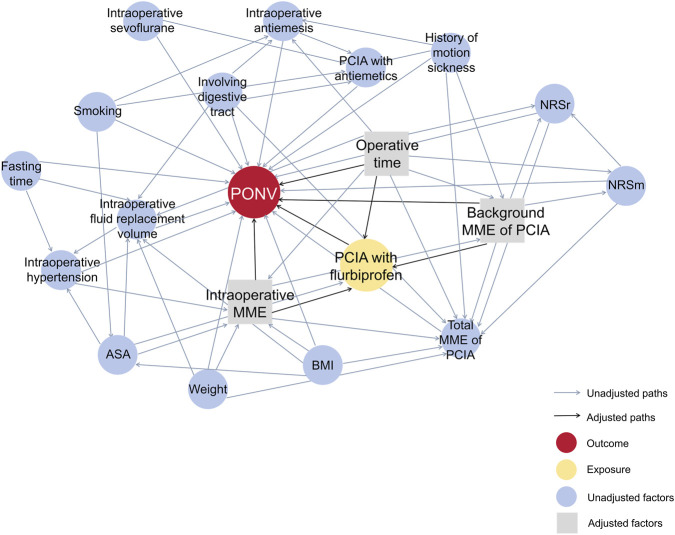
A directed acyclic graph represents associations between covariates and primary exposure and outcome. PONV, postoperative nausea and vomiting; BMI, body mass index; ASA, American Society of Anesthesiologists; PCIA, patient-controlled intravenous analgesia; MME, morphine milligram equivalents; NRSr, numeric rating scale at rest; NRSm, numeric rating scale during movement.

To elucidate the relationship between various perioperative factors and PONV, a directed acyclic graph (DAG) was constructed (Figure 2). The DAG allowed us to visually represent the causal structure and to identify potential confounders. The DAG implied that the following pre-treatment confounders required adjustment: operative time, intraoperative MME, background MME of PCIA.

Univariable logistic regression analysis identified eight significant perioperative variables associated with PONV (p < 0.05), including body weight, BMI, history of motion sickness or nausea and vomiting, intraoperative antiemesis, PCIA containing flurbiprofen, total MME of PCIA, NPRr and NPRm (Supplementary Table S1). Collinearity diagnostics among the eight variables revealed significant multicollinearity between body weight and BMI, with a variance inflation factor (VIF) > 5 (Supplementary Table S2). In accordance with established clinical parameters documented in prior literature, BMI was excluded and the remaining seven variables were incorporated into an unconditional binary logistic regression model (Supplementary Table S3). In the multivariable model, flurbiprofen-containing PCIA remained an independent predictor of PONV (OR = 1.412, 95% CI 1.042–1.914; p = 0.026). This reflects a 41% increase in the odds of PONV, which, at the observed baseline risk of 23%, equates to an absolute risk increase of ≈7% and an adjusted number needed to harm of ∼15 patients.

### 3.3 Sensitivity analysis

Several sensitivity analyses were conducted to verify the robustness of our findings. Propensity score matching was implemented to balance the patients whose PCIA containing flurbiprofen and the patients using flurbiprofen-free PCIA according to all perioperative characteristic covariates. After 1:1 matching, there were 416 patients in both groups (Table 2). As the standardized mean differences (SMD) < 0.1, all baseline characteristics were considered to be well-balanced. The difference of the incidence of PONV was still statistically significant after matching (23.0% vs. 30.3%, p = 0.041), the trend suggests that flurbiprofen-containing PCIA may contribute to an increased risk of PONV in gynecological laparoscopic surgery.

**TABLE 2:** Perioperative characteristics of the flurbiprofen-containing group and flurbiprofen-free group before and after propensity score matching.

​	Before matching			After matching			
Characteristics	PCIA without flurbiprofen (n = 430)	PCIA with flurbiprofen (n = 2000)	*p* value	PCIA without flurbiprofen (n = 416)	PCIA with flurbiprofen (n = 416)	SMD	*p* value
PONV	99 (23.0%)	554 (27.7%)	**0.047**	97 (23.0%)	123 (30.3%)	—	**0.041**
Age (yr)	47.5 (38.0, 55.0)	44.0 (36.0, 51.0)	**<0.001**	47.0 (38.0, 55.0)	47.0 (38.0, 54.0)	0.014	0.945
Weight (kg)	58.0 (51.3, 64.0)	56.2 (51.0, 63.0)	0.099	58.0 (51.4, 64.0)	57.1 (51.3, 63.0)	0.031	0.691
Height (cm)	158 (154, 161)	158 (155, 162)	0.066	158 (155, 161)	158 (155, 162)	0.020	0.705
BMI (kg/cm2)	23.1 (20.8, 25.6)	22.5 (20.5, 24.8)	**0.007**	23.1 (20.8, 25.6)	23.0 (20.9, 25.0)	0.041	0.561
History of motion sickness or nausea and vomiting	102 (23.7%)	498 (24.9%)	0.607	98 (23.7%)	102 (26.0%)	0.023	0.746
History of smoking	3 (0.7%)	14 (0.7%)	1.000	3 (0.7%)	4 (0.7%)	0.026	0.704
Hemoglobin (g/L)	121.5 (107.8, 130.0)	121.0 (107.0 130.0)	0.844	121.5 (107.8, 130.0)	119.0 (104.0, 128.0)	0.081	0.105
Albumin (g/L)	38.7 (36.5, 41.0)	39.2 (37.1, 42.0)	**0.007**	38.7 (36.5, 41.0)	38.6 (36.7, 41.3)	0.038	0.696
Fasting time (h)	14.08 (11.75, 16.27)	14.08 (11.75, 16.32)	0.382	14.09 (11.77, 16.33)	14.00 (11.79, 16.15)	0.017	0.821
ASA, n (%)	​	​	**<0.001**	​	​	0.045	0.811
I	47 (10.9%)	338 (16.9%)	​	47 (10.9%)	44 (14.0%)	​	​
II	310 (72.1%)	1,522 (76.1%)	​	309 (72.1%)	317 (72.9%)	​	​
III	73 (17.0%)	140 (7.0%)	​	60 (17.0%)	55 (13.1%)	​	​
Intraoperative sevoflurane	366 (85.1%)	1,638 (81.9%)	0.128	355 (85.1%)	359 (85.3%)	0.028	0.691
Intraoperative antiemesis	111 (25.8%)	703 (35.1%)	**<0.001**	110 (25.8%)	112 (29.0%)	0.011	0.876
Involving digestive tract	39 (9.1%)	199 (10.0%)	0.577	38 (9.1%)	40 (9.0%)	0.016	0.812
Operative time (h)	2.63 (1.93, 3.92)	2.73 (2.00, 3.92)	0.936	2.59 (1.92, 3.83)	2.75 (2.00, 3.91)	0.044	0.420
Intraoperative hypotension	369 (85.8%)	1,650 (82.5%)	0.096	356 (85.8%)	354 (83.1%)	0.014	0.845
Intraoperative MME (mg/kg)	2.58 (1.86, 4.07)	2.77 (1.90, 3.98)	0.237	2.56 (1.85, 3.98)	2.67 (1.80, 4.02)	0.005	0.964
Intraoperative fluid replacement volume (mL/kg)	24.49 (18.07, 33.84)	24.49 (18.46, 32.89)	0.976	24.22 (17.94, 33.33)	24.47 (18.33, 33.96)	0.045	0.614
Intraoperative blood loss (mL/kg)	0.76 (0.31, 1.54)	0.82 (0.33, 1.75)	0.198	0.74 (0.30, 1.53)	0.85 (0.33, 1.72)	0.062	0.091
Intraoperative urine output (mL/kg)	1.46 (0, 5.17)	1.04 (0, 4.53)	0.066	1.27 (0, 5.02)	1.42 (0, 4.76)	0.025	0.916
Intraoperative net fluid balance (mL/kg)	20.55 (14.38, 27.38)	20.89 (15.32, 28.21)	0.779	20.37 (14.29, 27.17)	21.23 (15.39, 28.56)	0.034	0.274
P_ET_CO_2_ < 30 mmHg (minutes)	20 (5, 65)	20 (5, 70)	0.353	20 (5, 65)	30 (5, 98.75)	0.114	0.118
P_ET_CO_2_ > 40 mmHg (minutes)	0 (0, 5)	0 (0, 5)	0.999	0 (0, 5)	0 (0, 5)	0.089	0.619
PCIA containing antiemetics	78 (18.1%)	302 (15.1%)	0.115	76 (18.1%)	72 (18.3%)	0.025	0.717
Background MME of PCIA (μg/kg/h)	7.85 (3.18, 14.17)	6.09 (0.00, 11.01)	**<0.001**	7.74 (3.07, 13.78)	6.77 (0.42, 12.55)	0.010	0.094
Total MME of PCIA (μg/kg)	394.6 (189.1, 746.3)	313.8 (149.0, 624.9)	**0.001**	389.7 (185.2, 719.2)	335.6 (162.1, 667.5)	0.018	0.123
Postoperative LHS (days)	5 (4, 6)	5 (3, 6)	0.537	5 (4, 6)	5 (4, 6)	0.021	0.493
NRSr, M (Q1, Q3)	0 (0, 1)	0 (0, 1)	0.674	0 (0, 1)	0 (0, 1)	0.061	0.406
NRSm, M (Q1, Q3)	1 (1, 2)	2 (1, 2)	0.677	1 (1, 2)	2 (1, 2)	0.053	0.586

Values given as Mdn (IQR) or n (%).

Abbreviations: PCIA, patient-controlled intravenous analgesia; SMD: standardized mean difference; BMI, body mass index; ASA, american society of anesthesiologists; MME, morphine milligram equivalents; Intraoperative net fluid balance = Intraoperative fluid replacement volume - Intraoperative blood loss - Intraoperative urine output, expressed per kilogram of body weight; P_ET_CO_2_, End-tidal carbon dioxide partial pressure; LHS, length of hospital stay; NRSr, numeric rating scale at rest; NRSm, numeric rating scale during movement.

**FIGURE 3:**
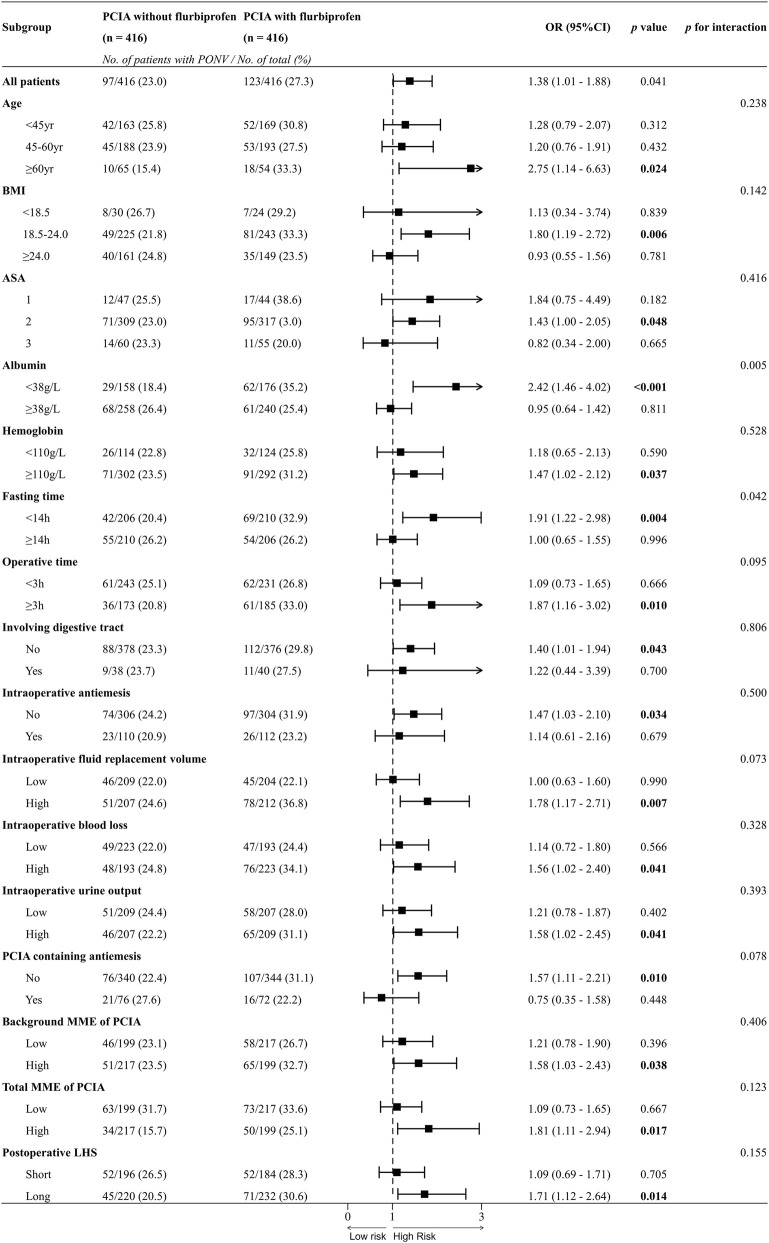
Forest plot of subgroup analysis. OR, odds ratio; CI, confidence interval; PCIA, patient-controlled intravenous analgesia; BMI, body mass index; ASA, American Society of Anesthesiologists; MME, morphine milligram equivalents; LHS, length of hospital stay.

In exploratory subgroup analyses stratified by different variables, the association between flurbiprofen-containing PCIA and PONV remained significant in the groups which have older age (>60 years), normal range of BMI (18.5–24.0 kg/cm^2^), lower albumin level (<38 g/L), normal range of hemoglobin (≥110 g/L), shorter fasting time (<14 h), longer operative time (>3 h), no involving digestive tract, no using intraoperative antiemesis, higher intraoperative fluid replacement volume, higher intraoperative blood loss, higher intraoperative urine output, no using PCIA antiemesis, longer postoperative LHS, using high dosage of background MME and total MME of PCIA (Figure 3).

### 3.4 Exploratory analysis

**FIGURE 4:**
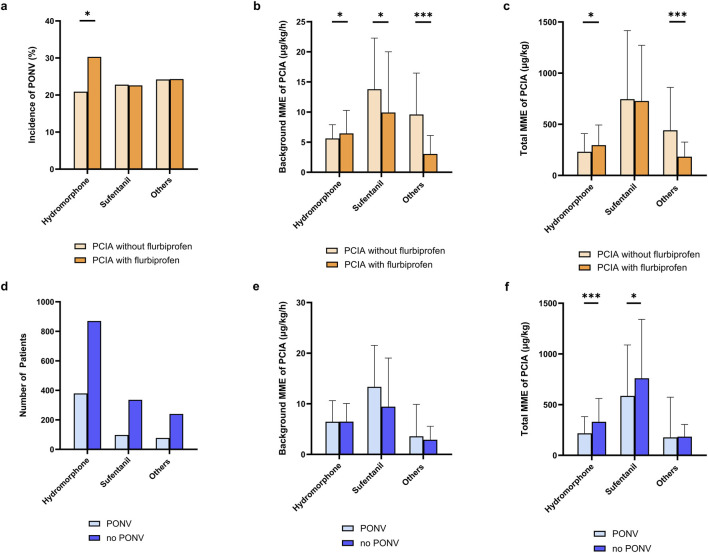
Exploratory analysis of the patients using various opioids combined with flurbiprofen in PCIA. Bar charts depict median values with upper quartile. **(a)** incidence of PONV with different opioid regimens with or without flurbiprofen; **(b)** background consumption of MME in PCIA across different regimens; **(c)** total consumption of MME in PCIA across different regimens; **(d)** numbers of patients using flurbiprofen-based PCIA with different opioid formulas; **(e)** background consumption of MME in flurbiprofen-based PCIA between patients with and without PONV across different regimens; **(f)** total consumption of MME in flurbiprofen-based PCIA between patients with and without PONV across different regimens.

In addition to the incidence of PONV, we also examined the association between formulas and opioid consumption of PCIA. The incidence of PONV with different opioids regimen in PCIA is shown in Figure 4a. Interestingly, when hydromorphone was combined with flurbiprofen, the incidence of PONV was higher compared with hydromorphone alone (30.3% vs. 20.9%; OR 1.646, 95% CI 1.059–2.558, p = 0.026), and both background MME [6.46 (1.08–10.28) vs. 5.62 (2.47–7.91) μg/kg/h, p = 0.023] and postoperative PCIA total MME [296.8 (152.4–493.4) vs. 231.3 (145.0–409.2) μg/kg, p = 0.035] were significantly higher (Figures 4b,c).

Furthermore, when focusing only on patients receiving flurbiprofen-containing PCIA, the distribution of patients receiving different opioid formulations combined with flurbiprofen is presented in Figure 4d. The incidence of PONV was significantly higher in those using hydromorphone compared with sufentanil (30.3% vs. 22.6%; OR 1.49, 95% CI 1.157–1.928, p = 0.002). Meanwhile, the hydromorphone group had lower background MME [6.46 (1.08–10.28) vs. 9.93 (0.00–20.00) μg/kg/h, p < 0.001] and lower postoperative PCIA total MME [296.8 (152.4–493.4) vs. 727.5 (322.9–1,272.0) μg/kg, p < 0.001]. In addition, regardless of whether hydromorphone or sufentanil was used, patients who developed PONV had lower PCIA total MME compared with those who did not. These findings suggest that both the choice of opioid and its combination with flurbiprofen may influence the incidence of PONV as well as postoperative opioid consumption patterns.

## 4 Discussion

In this retrospective cohort of 2,430 patients undergoing gynecologic laparoscopy, postoperative nausea and vomiting (PONV) occurred in 26.9%. The use of flurbiprofen in patient-controlled intravenous analgesia (PCIA) was independently associated with higher PONV risk after adjustment for confounders. This association was most pronounced in older patients and those with longer operation time of PCIA and other subgroups. Meanwhile, flurbiprofen-containing PCIA was also associated with lower postoperative morphine milligram equivalents (MME). Notably, flurbiprofen combined with hydromorphone was associated with higher PONV incidence and increased PCIA MME relative to hydromorphone alone. In summary, while flurbiprofen-containing PCIA was observed to be associated with opioid-sparing benefits, this potential analgesic advantage was accompanied by an increased risk of PONV in our cohort. Importantly, given the retrospective observational design, residual confounding and indication bias cannot be fully excluded, and these findings demonstrate an association rather than establishing causation.

Our results highlight a complex balance between opioid-sparing benefits and emetogenic risks when incorporating non-steroidal anti-inflammatory drugs (NSAIDs) into postoperative analgesia. Enhanced Recovery After Surgery (ERAS) protocols advocate multimodal analgesia (including NSAIDs) to minimize opioid use and improve recovery (Gan et al., 2020; Cozowicz et al., 2024; Steiness et al., 2024). Consistent with this principle, flurbiprofen use in our cohort was associated with reduced postoperative opioid consumption. However, this reduction in MME did not correspond to a lower incidence of PONV. Typically, reducing opioid consumption is associated with lower PONV rates, NSAIDs-based analgesia has been shown to decrease PONV incidence (Smith et al., 2012; Lee et al., 2023). In contrast, our findings suggest that the potential antiemetic benefit of opioid reduction may not fully offset other effects associated with flurbiprofen in this specific context. These results underscore that opioid-sparing strategies do not uniformly translate into lower PONV rates.

One possible explanation involves flurbiprofen’s pharmacological effects on the gastrointestinal system. NSAIDs inhibit cyclooxygenase and thereby prostaglandin synthesis. Prostaglandins play roles in maintaining gastric mucosal integrity and modulating gastrointestinal motility. It is biologically plausible that the prostaglandin inhibition effect of NSAIDs may influence gastric emptying, visceral sensitivity, or central emetic signaling pathways, potentially contributing to nausea and vomiting (Zhong et al., 2021; Bindu et al., 2020). Thus, an opioid-sparing strategy may not reduce PONV if the adjuvant analgesic (flurbiprofen in this case) induces PONV *via* other mechanisms. However, these mechanistic interpretations remain speculative. Our study did not directly measure gastric motility, prostaglandin levels, or central emetic mediators. Therefore, the proposed pathways should be considered hypothesis-generating rather than definitive explanations. Future mechanistic studies are warranted to clarify whether prostaglandin inhibition directly contributes to PONV in this clinical setting.

We also observed that patients who experienced PONV had higher postoperative pain scores, indicating a bidirectional relationship between pain and nausea. Inadequate pain control may exacerbate nausea, while nausea and vomiting may limit effective PCIA use, leading to suboptimal analgesia (Yaşlı et al., 2024). This interplay reinforces the importance of optimizing pain management and antiemetic prophylaxis in the postoperative period (Elvir-Lazo et al., 2020). Multimodal strategies should address both and PONV concurrently, rather than focusing on one alone.

Older patients (>60 years) in our cohort had a greater increase in PONV risk with flurbiprofen, contrary to the conventional view that younger age is a strong risk factor for PONV (Apfel et al., 2012). One explanation is that elderly patients have reduced physiological reserves. The steep Trendelenburg position used in laparoscopy can increase intracranial pressure; older individuals may tolerate this poorly (Bindu et al., 2020; Robba et al., 2016), and combined with NSAIDs-related gastric stress, the threshold for triggering PONV could be lowered in this group. Age-related vulnerability might amplify flurbiprofen’s emetogenic effects, even though baseline PONV risk is generally lower in older patients. Nevertheless, subgroup findings should be interpreted cautiously. Given the observational design and smaller sample sizes within certain strata, these results may be influenced by residual confounding and should be considered exploratory.

Our subgroup findings further suggest that the choice of opioid in a multimodal regimen was associated with net PONV outcomes. Despite the inherent heterogeneity in PCIA formulations, the adjusting for MME and stratifying by opioid type allowed us to isolate the potential interaction between flurbiprofen and specific opioids. When flurbiprofen was combined with hydromorphone, the incidence of PONV increased compared with hydromorphone alone. In addition, both background and total opioid doses were higher. This finding suggests that concomitant use of NSAIDs may not provide the expected opioid-sparing benefit in combination with specific opioids. Besides, flurbiprofen combined with hydromorphone was associated with significantly more PONV than flurbiprofen with sufentanil, implying hydromorphone may exacerbate PONV when paired with an NSAID. A possible explanation is that hydromorphone generates metabolites with broader receptor engagement and emetogenic potential, while sufentanil lacks such metabolites (Wehrfritz et al., 2022). In contrast, sufentanil’s higher potency allows effective analgesia at lower doses, potentially causing less nausea (Koo et al., 2024; Liu et al., 2022). This finding suggests that concomitant use of NSAIDs may not provide the expected opioid-sparing benefit in combination with specific opioids, highlighting the importance of considering regimen composition in future studies.

This study offers clinically relevant insights into NSAIDs-based multimodal analgesia and PONV in minimally invasive gynecologic surgery. It is one of the largest investigations of flurbiprofen’s impact on PONV. We employed rigorous methods to minimize bias, including a directed acyclic graph (DAG) for confounder selection, propensity score matching, and multivariable logistic regression. Additionally, detailed subgroup analyses (e.g., stratification by opioid type) generated valuable hypotheses regarding opioid–NSAIDs interactions and PONV management.

However, several limitations must be acknowledged. As a retrospective single-center study, there is a risk of selection bias and residual confounding. Our data were derived from electronic medical records, which may vary in PONV assessment and documentation. Specifically, PONV was assessed using a simplified NVDS scale without differentiation between nausea and vomiting, precise timing of onset, or the administration of rescue antiemetics. We lacked detailed information on these granular aspects of nausea severity and temporal patterns, which may obscure clinically meaningful differences and represents a limitation of our retrospective design. Second, the analgesic regimens were not randomized. Thus, indication bias in the choice to include NSAIDs is possible. Although we employed DAG-guided confounder selection and propensity score matching to minimize bias, unmeasured clinical factors (e.g., anticipated pain severity influencing the decision to include flurbiprofen) may still exist. Therefore, while flurbiprofen use was significantly associated with PONV, causation cannot be established in this observational study. Future randomized controlled trials are needed to verify whether this association reflects a causal relationship. Prospective confirmation is needed, ideally through randomized controlled trials (RCTs) comparing opioid-only vs. opioid + NSAIDs PCIA, to verify causality and elucidate mechanisms.

In summary, this large retrospective study demonstrated that while flurbiprofen-containing PCIA was associated with reduced postoperative opioid requirements, it is paradoxically associated with the increased risk of PONV. The emetogenic effect was most evident in elderly patients and in those receiving hydromorphone-based PCIA. These results highlight a trade-off between analgesic benefit and PONV risk when incorporating NSAIDs into multimodal analgesia.

Clinically, our findings suggest that flurbiprofen should not be considered a universally benign adjunct. Instead, its use should be individualized based on each patient’s baseline PONV risk and the choice of co-administered opioid. In particular, caution is warranted when flurbiprofen is paired with hydromorphone, where both opioid consumption and PONV incidence were increased. Enhanced antiemetic prophylaxis or alternative analgesic strategies may be preferable for these high-risk subgroups.

## 5 Conclusion

In conclusion, this retrospective analysis identified an independent association between the addition of flurbiprofen to PCIA and a higher incidence of PONV in patients undergoing gynecologic laparoscopic surgery. While causality cannot be established from this observational study, the findings suggest that flurbiprofen should not be considered a universally benign adjunct. Prospective randomized studies are warranted to confirm causality and refine multimodal analgesia protocols.

## Data Availability

The raw data supporting the conclusions of this article will be made available by the corresponding authors, without undue reservation.
