# Supplementary material for: Flurbiprofen in patient-controlled intravenous analgesia and the risk of postoperative nausea and vomiting after gynecologic laparoscopy: a retrospective case-control study
Source: Front Pharmacol. 2026 Mar 19;17:1785675. doi: 10.3389/fphar.2026.1785675 (PMC13044099; doi:10.3389/fphar.2026.1785675)
Supplement: Supplementary file 1 [file Table1.docx]

**Supporting information**

**Table S1 Univariate regression analysis of clinical characteristics**

| Variables | | | OR | 95%CI | *p* value |
| --- | --- | --- | --- | --- | --- |
| Age (years) | | | 0.995 | 0.988-1.003 | 0.244 |
| Weight (kg) | | | 0.980 | 0.970-0.990 | **<0.001** |
| Height (cm) | | | 0.990 | 0.974-1.007 | 0.238 |
| BMI (kg/cm2) | | | 0.953 | 0.927-0.979 | **<0.001** |
| History of motion sickness or nausea and vomiting | | | 1.427 | 1.167-1.745 | **<0.001** |
| History of smoking | | | 0.581 | 0.166-2.029 | 0.395 |
| Hemoglobin (g/L) | | | 1.004 | 0.999-1.009 | 0.088 |
| Albumin (g/L) | | | 1.007 | 0.985-1.031 | 0.528 |
| Fasting time (hours) | | | 1.019 | 0.988-1.050 | 0.232 |
| ASA, n (%) | | |  |  |  |
| Ⅰ | | Ref. | Ref. | Ref. |  |
| Ⅱ |  | 1.022 | 0.798-1.310 | 0.863 |  |
| Ⅲ |  | 0.884 | 0.601-1.300 | 0.532 |  |
| Intraoperative sevoflurane | | | 1.100 | 0.866-1.397 | 0.436 |
| Intraoperative antiemesis | | | 0.600 | 0.491-0.733 | **<0.001** |
| Involving digestive tract | | | 1.230 | 0.919-1.646 | 0.164 |
| Operative time (hours) | | | 1.019 | 0.963-1.078 | 0.515 |
| Intraoperative hypotension | | | 1.086 | 0.852-1.383 | 0.506 |
| Intraoperative MME (mg/kg) | | | 1.009 | 0.968-1.052 | 0.668 |
| Intraoperative fluid replacement volume  (mL/kg) | | | 1.006 | 1.000-1.011 | 0.050 |
| Intraoperative blood loss (mL/kg) | | | 1.021 | 0.995-1.047 | 0.117 |
| Intraoperative urine output (mL/kg) | | | 1.006 | 0.989-1.023 | 0.505 |
| Intraoperative net fluid balance (mL/kg) | | | 1.007 | 1.000-1.015 | 0.066 |
| P_ET_CO_2_ < 30mmHg (minutes) | | | 0.999 | 0.998-1.000 | 0.091 |
| P_ET_CO_2_ > 40mmHg (minutes) | | | 1.002 | 0.999-1.005 | 0.130 |
| PCIA containing flurbiprofen | | | 1.281 | 1.003-1.636 | **0.048** |
| PCIA containing antiemetics | | | 0.806 | 0.624-1.041 | 0.099 |
| Background MME in PCIA (ug/kg/hour) | | | 1.005 | 0.997-1.014 | 0.226 |
| Total MME of PCIA (ug/kg) | | | 0.999 | 0.999-0.999 | **<.001** |
| Postoperative LHS (days) | | | 1.008 | 0.982-1.035 | 0.551 |
| NRSr, M (Q1, Q3) | | | 4.000 | 3.505-4.565 | **<.001** |
| NRSm, M (Q1, Q3) | | | 2.683 | 2.433-2.959 | **<.001** |

Abbreviations: BMI, body mass index; ASA, American Society of Anesthesiologists; MME, morphine milligram equivalents; P_ET_CO_2_, End-tidal carbon dioxide partial pressure; PCIA, patient-controlled intravenous analgesia; LHS, length of hospital stay; NRSr, numeric rating scale at rest; NRSm, numeric rating scale during movement

**Table S2** **Diagnosis of collinearity.**

| Variables | Tolerance | VIF |
| --- | --- | --- |
| Weight | 0.191 | 5.242 |
| BMI | 0.188 | 5.312 |
| History of motion sickness or nausea and vomiting | 0.995 | 1.005 |
| Operative time | 0.434 | 2.306 |
| Intraoperative antiemesis | 0.976 | 1.024 |
| Intraoperative MME | 0.450 | 2.224 |
| Background MME of PCIA | 0.674 | 1.483 |
| Total MME of PCIA | 0.654 | 1.529 |
| PCIA containing flurbiprofen | 0.981 | 1.020 |
| NPRr | 0.304 | 3.293 |
| NPRm | 0.300 | 3.332 |

Abbreviations: VIF, variance inflation factor; BMI, body mass index; MME, morphine milligram equivalents; PCIA, patient-controlled intravenous analgesia; NRSr, numeric rating scale at rest; NRSm, numeric rating scale during movement

**Table S3 Results of multivariable logistic regression**

| Variables | OR | 95%CI | *p* value |
| --- | --- | --- | --- |
| Weight | 0.976 | 0.964-0.989 | **< 0.001** |
| History of motion sickness or nausea and vomiting | 0.278 | 0.052-1.491 | **0.135** |
| Operative time | 0.995 | 0.887-1.118 | 0.937 |
| Intraoperative antiemesis | 0.510 | 0.398-0.654 | **< 0.001** |
| Intraoperative MME | 1.003 | 0.916-1.098 | 0.946 |
| Background MME of PCIA | 1.044 | 1.028-1.060 | **< 0.001** |
| Total MME of PCIA | 0.999 | 0.998-0.999 | **< 0.001** |
| PCIA containing flurbiprofen | 1.412 | 1.042-1.914 | **0.026** |
| NPRr | 2.507 | 2.006-3.134 | **< 0.001** |
| NPRm | 1.776 | 1.507-2.092 | **< 0.001** |

The multivariable logistic regression was based on variables selected using the directed acyclic graph and variables with a P-value less than 0.05 in univariate logistic regression analyses.

Abbreviations: MME, morphine milligram equivalents; PCIA, patient-controlled intravenous analgesia; NRSr, numeric rating scale at rest; NRSm, numeric rating scale during movement

**Table S4 Types of combined opioids in PCIA and PONV incidence of different formulas.**

| Combined opioids | PCIA with flurbiprofen | | | PCIA without flurbiprofen | | |  |  |  |
| --- | --- | --- | --- | --- | --- | --- | --- | --- | --- |
|  | Total patients (n = 2000) | PONV  (n = 554) | PONV incidence (%) | Total patients (n = 430) | PONV  (n = 99) | PONV incidence (%) | *p* value | OR | 95% CI |
| Hydromorphone | 1249 | 379 | 30.3 | 129 | 27 | 20.9 | 0.026 | 1.646 | 1.059-2.558 |
| Sufentanil | 434 | 98 | 22.6 | 57 | 13 | 22.8 | 0.969 | 0.987 | 0.511-1.907 |
| Oxycodone | 156 | 46 | 29.5 | 5 | 2 | 40.0 | 0.635 | 0.627 | 0.101-3.879 |
| Butorphanol | 50 | 9 | 18.0 | 0 | 0 | - | - | - | - |
| Butorphanol +  Hydromorphone | 85 | 17 | 20.0 | 166 | 48 | 28.9 | 0.127 | 0.615 | 0.328-1.152 |
| Butorphanol +  Sufentanil | 25 | 5 | 20.0 | 69 | 9 | 13.0 | 0.512 | 1.667 | 0.500-5.559 |
| Butorphanol +  Oxycodone | 0 | 0 | - | 4 | 0 | 0.0 | - | - | - |
| No opioids | 1 | 0 | 0.0 | 0 | 0 | - | - | - | - |

**Table S5 Background MME and Total MME of PCIA across different regimens.**

| Combined opioids | Background MME of PCIA (μg/kg/h) | | | Total MME of PCIA (μg/kg) | | |
| --- | --- | --- | --- | --- | --- | --- |
|  | PONV (n = 554) | No PONV (n = 1446) | *p* value | PONV (n = 554) | No PONV (n = 1446) | *p* value |
| Hydromorphone | 6.46 (0.00, 10.61) | 6.48 (2.82, 10.06) | 0.923 | 217.1 (101.7, 381.6) | 331.0 (190.6, 560.7) | **<0.001** |
| Sufentanil | 13.36 (0.00, 21.53) | 9.43 (0.00, 19.03) | 0.108 | 586.0 (229.2, 1088.0) | 759.7 (351.8, 1341.0) | **0.028** |
| Other regimens | 3.57 (0.00, 9.88) | 2.88 (0.00, 5.55) | 0.111 | 176.6 (61.0, 573.4) | 184.4 (95.6, 304.3) | 0.793 |

**Table S6 Background MME and Total MME of flurbiprofen-based PCIA between patients with and without PONV across different regimens**

| Combined opioids | Background MME of PCIA (μg/kg/h) | | | Total MME of PCIA (μg/kg) | | |
| --- | --- | --- | --- | --- | --- | --- |
|  | without flurbiprofen  (n = 430) | with flurbiprofen  (n = 2000) | *p* value | without flurbiprofen  (n = 430) | with flurbiprofen  (n = 2000) | *p* value |
| Hydromorphone | 5.62 (2.47, 7.91) | 6.46 (1.08, 10.28) | **0.023** | 231.3 (145.0, 409.2) | 296.8 (152.4, 493.4) | **0.035** |
| Sufentanil | 13.79 (5.77, 22.27) | 9.93 (0.00, 20.00) | **0.034** | 746.0 (472.8, 1415.8) | 727.5 (322.9, 1272.0) | 0.388 |
| Other regimens | 9.59 (4.04, 16.46) | 3.04 (0.00, 6.11) | **<0.001** | 441.3 (222.0, 861.0) | 183.6 (91.9, 326.9) | **<0.001** |
